# PFAPA syndrome is linked to dysregulated IL-1β production

**DOI:** 10.1186/1546-0096-9-S1-P297

**Published:** 2011-09-14

**Authors:** Laeticia Kolly, Annette von Scheven-Gete, Nathalie Busso, Nathaliane Bagnoud, Dirk Holzinger, Michael Morris, Michael Hofer

**Affiliations:** 1 Service of rheumatology, DAL, CHUV, Lausanne, Switzerland; 2 Pediatric rheumatology romande, DMCP, CHUV, Lausanne and HUG, Genève, Switzerland; 3 University hospital of Munster, Germany; 4 Service of genetic medicine, HUG, Genève, Switzerland

## Background

Auto-inflammatory diseases (AID) are characterized by recurrent or chronic febrile episodes due to a defect in the regulation of the inflammatory response linked to the innate immunity. For some AID, a monogenic origin has been established recently. The genetic defect is related or is suspected to be related to a molecular complex called inflammasome, which is involved in the regulation of IL-1β, a cytokine with a central role in innate immunity. The inflammasome activates the proinflammatory caspase-1, which then cleaves the pro-IL-1β to IL-1β. PFAPA syndrome is characterized by recurrent fever associated with aphtosis, pharyngitis, and cervical adenitis with a spontaneous resolution in most cases until adolescence. There is no clear aetiology found up to now and recently a familial predominance has been shown suggesting a genetic cause.

## Aim

Evaluate the role of dysregulation of IL-1β secretion in the pathogenesis of PFAPA syndrome.

## Methods

In 12 patients with confirmed PFAPA syndrome blood was drawn during (IN) and outside a febrile episode (OUT). Blood count, erythrocytes sedimentation rate (ESR), C-reactive protein (CRP), serum amyloid A (SAA), cytokines levels,MRP8/14 and S100A12 (proteins secreted by granulocytes and monocytes) were measured. PBMCs were isolated using a Ficoll-Paque gradient and stimulated with LPS in the presence or not of ZYVAD, a caspase inhibitor. IL-1β levels were dosed in the supernatants by ELISA and active IL-1β was visualized by Western-blot. Genomic DNA was screened by PCR and sequencing for genetic variants of MEFV, TNFRSF1A, MVK and NLRP3 genes.

## Results

Monocytes and neutrophiles counts, ESR, CRP and SAA levels were significantly elevated during febrile episodes as well as MRP8/14 and S100A12. PBMCs secreted more IL-1β (active p17IL-1β form) upon LPS stimulation during fever flares (p<0.001: OUT 235+/-56 pg/ml; IN 575+/-88 pg/ml). The secretion of IL-1β could be inhibited by ZYVAD, a caspase inhibitor. Serum levels of IL-6, IP-10 and caspase -1 were also increased significantly during febrile episode whereas TNFα and MCP-1 did not show significant changes. 4 of 12 patients were found to have a heterogeneous variant in the NLRP3 gene but no variant in the MEFV, TNFRSF1A and MVK genes. These patients did not have significantly different symptoms of their PFAPA syndrome in comparison to the patients without the variants.

## Conclusion

In PFAPA patients, stimulated mononuclear cells have an increased IL-1β secretion during a fever episode dependant on a caspase-1 inflammasome which suggests a role for IL-1β in the pathogenesis of PFAPA. Interestingly, NLRP3 mutations were found in a subset of patients, which could be associated with gain-of-function mutations of the NLRP3 gene. These findings may open new treatment options in PFAPA.

